# Identification and Characterization of AUXIN Response Factor Gene Family Reveals Their Regulatory Network to Respond the Multi-Hormones Crosstalk during GA-Induced Grape Parthenocarpic Berry

**DOI:** 10.3390/ijms231911108

**Published:** 2022-09-21

**Authors:** Zilu Sheng, Xuxian Xuan, Fei Wang, Ehsan Sadeghnezhad, Peijie Gong, Yingke Xiao, Tianyu Dong, Peian Zhang, Xicheng Wang, Jinggui Fang, Chen Wang

**Affiliations:** 1 College of Horticulture, Nanjing Agricultural University, Nanjing 210095, China; 2 Department of Plant Biology, Faculty of Biological Science, Tarbiat Modares University, Tehran 843300, Iran

**Keywords:** grape, auxin response factors, gibberellin, parthenocarpy

## Abstract

Exogenous gibberellin (GA) was widely used to improve berry quality through inducing parthenocarpic seedless berries in grapes. We revealed that auxin response factors (ARFs), the key transcription factors in response to auxin, might respond to GA involving modulation of grape parthenocarpy. However, the underlying molecular mechanism in this process remains yet unclear. Here, a total of 19 VvARF members were identified in the ovaries during GA-induced grapes’ parthenocarpy. Interestingly, almost all members were GA-responsive factors, of which 9 could be classified in plant hormone signal transduction (KO04075) and involved in the tryptophan metabolic pathway (K14486). Moreover, VvARFs were predicted to have 310 interacted proteins involved in 19 KEGG pathways. Of them, 32 interacted proteins participated in the KO04075 pathway, including auxin (IAA), salicylic acid (SA), abscisic acid (ABA), cytokinin (CTK), and ethylene signaling pathways by responding to GA-mediated multi-hormone crosstalk. Further analysis demonstrated that *VvARF4-2* might be the major factor in the modulation of GA-induced parthenocarpy via the crosstalk of IAA, CTK, SA, and ethylene signaling, followed by *VvARF6-1* and *VvARF9* involved in SA and ABA signaling pathways, respectively. Finally, we developed a VvARFs-mediated regulatory network by responding to GA-mediated multi-hormone crosstalk during grape parthenocarpy. Collectively, our findings provided novel insights into the regulatory network of VvARFs in GA-guided multi-hormone signaling to modulate grape parthenocarpy, which has great implications for the molecular breeding of high quality seedless grape berries.

## 1. Introduction

Gibberellin (GA) is an essential phytohormone, which plays important roles in multiple aspects of plant growth and development, such as seed germination, stem hypocotyl elongation, leaf elongation, cell expansion, epidermal hair development, controlling flowering time, and flower and fruit development, especially in parthenocarpy [1]. Nowadays, exogenous GA is widely used to improve berry quality through inducing parthenocarpic seedless berries in grape, kiwi fruit, loquat, pear, cherry, litchi, and other species [2,3]. Meanwhile, the crosstalk between GA and auxin signaling is necessary for fruit initiation and development [4]. Correspondingly, GA- and auxin-signaling components can trigger parthenocarpy and contribute to seedless fruit production. Although, auxin and GA act in fruit growth and parthenocarpy induction, with distinct functions [5].

The understanding of molecular mechanisms underlying auxin-regulated processes in collaboration with GA-signaling cascades is required for creating seedless fruits. Previous studies showed that several members of the auxin response factors (ARFs) family could be involved in parthenocarpy [6]. Moreover, ARFs as pivotal transcription factors regulate the expression of auxin responsive genes through binding to auxin response cis-acting elements (AuxREs) [7]. Most ARF members contain three domains: DBD (DNA- binding Domain), MR (Middle Region), and CTD (C-terminal domain) [8]. The DBD region of the ARF protein can bind to the TGTCTC sequence of the AuxRE element [9]. MR domain is a central functional region with transcriptional activation or inhibition activity, and CTD domain is involved in the binding of ARFs to AuxRE and regulating the expression of downstream auxin response genes [10]. In *Solanum lycopersicum* (tomato), miR167/*ARF8* and miR159/*GAMYB* act synergistically to regulate tomato ovule development and produce parthenocarpic seedless fruits [11]. The expression of aberrant *ARF8* stimulates parthenocarpy in *Arabidopsis thaliana* and tomato [12], while the GA-signaling repressor *SlDELLA* and auxin-signaling components *SlIAA9* and *SlARF7* repress the parthenocarpy in tomato [13]. Similarly, our previous study also revealed that exogenous GA could distinctly up-regulate the expression of *VvARF10(VIT_208s0040g01810)/16(VIT_213s0019g04380)/17*(*VIT_218s0001g04180*) accompanied with the induction of grape parthenocarpy [6]. These demonstrate some ARF members respond to auxin and GA signaling [14,15] and greatly function during the GA-induced parthenocarpy process.

There is a different number of ARFs members among plant species. With the improvement of genome sequencing technology, 25, 23, 37, 29, and 21 members of ARF gene’s family have been identified in *Oryza sativa*, *Arabidopsis thaliana*, *Populus trichocarpa, Malus domestic,* and *Solanum lycopersicum*, respectively [16,17,18,19,20]. Meanwhile, ARFs were characterized to be involved in the modulation of leaf senescence, flowering, abscission, fruit, and other tissue development [21,22,23]. For example, *ARF2* gene is induced and expressed in senescent leaves, regulating the time of leaf senescence, flowering, and abscission in *Arabidopsis thaliana* [24]. In addition, *SlARF6* and *SlARF8* mainly regulate the development process of tomato flowers and fruits [13], and *AtARF8* can also regulate the elongation of *Arabidopsis thaliana* hypocotyls [25]. Ectopic expression of auxin glycosyltransferase UGT84A2 in *Arabidopsis thaliana* can delay flowering through increasing indole-3-butyric acid and suppressing the transcription of *ARF6*, *ARF8,* and flowering-related genes *FT*, *SOC1*, *AP1*, and *LFY* [26]. Moreover, *SlARF* not only mediates part of auxin signaling, but also acts as part of gibberellin signaling during the development of tomato fruits [27,28]. The phytohormones brassinosteroid (BR), auxin, and gibberellin regulate photomorphogenesis-related hypocotyl elongation in arabidopsis via the cooperative interaction of BZR-ARF-PIF/DELLA (BAP/D) transcription factors/regulators [29]. Moreover, the interaction between the CpARF2 and CpEIL1 mediates the interaction between auxin and ethylene signaling to regulate fruit ripening in papaya [30]. All these studies confirm that the various members belonging to the ARF family provide the diversification of functions across plant species, which synchronize plant growth and development of fruits based on the hormone-dependent signaling.

It seems that GA-induced parthenocarpy in grape is more typical than other fruit trees. Grape, as an important economical fruit crop in the world, can be used in human diets such as fresh berry, juice, and wine. Therefore, seedless grapes are very popular with consumers. However, due to the shortage of seedless grape varieties, at present, GA-induced parthenocarpy is the main approach of producing seedless berries in the grape industry. Nevertheless, the mechanism by which GA modulates parthenocarpy in collaboration with the ARF gene family is poorly understood. In this study, we characterized the ARF gene family at the whole genome level and investigated the ARFs-mediated regulatory network via multi-hormone signal crosstalk during the GA-induced parthenocarpy process in grapes. Our findings provide important insights into how VvARFs’ regulatory network promotes the parthenocarpic process in grape inflorescences, leading to significant implications for the development of high-quality grape seedless berries.

## 2. Results

### 2.1. Changes of Berry Development and Quality during GA-Induced Parthenocarpy in Grapes

**Figure 1:**
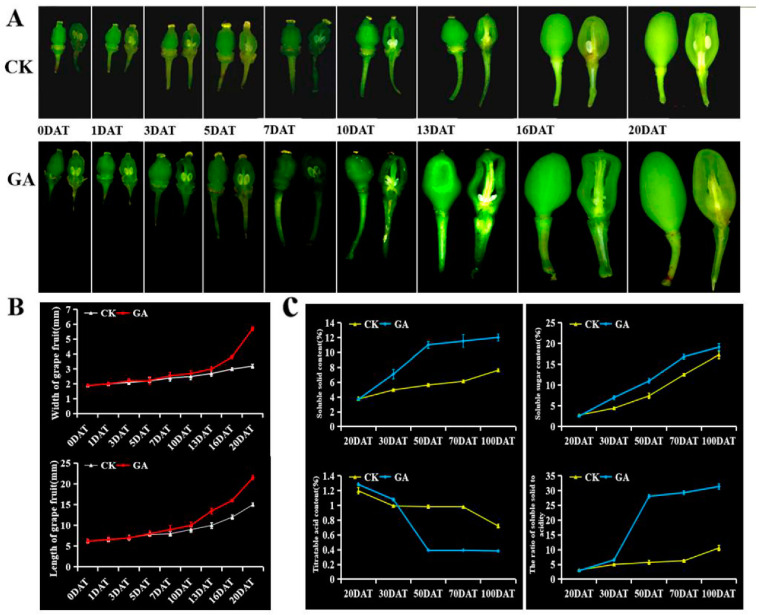
Morphological changes of berries development during grapevine parthenocarpy process-induced by exogenous gibberellin (GA) application. (**A**). Morphological changes of ‘Wink’ cultivar during berries development in response to GA_3_ treatment at different time points [0, 1, 3, 5, 7, 10, 13, 16, and 20 days aftertreatment (DAT)]. (**B**). Effect of GA_3_ on the length and width of grape fruits. (**C**). Effects of GA treatment on soluble solids and titratable acid content of grape berries at different time points [20, 30, 50, 70, and 95 days aftertreatment (DAT)].

We evaluated the ‘Wink’ grape berry development in a time course behavior in response to GA_3_ treatment. After 10 DAT (Days after treatment) with GA_3_, the development of their seeds was repressed drastically and led to seedless with a rate of 99.6%, while their berry brushes had no obvious changes (Figure 1A). In contrast, the seeds in control group without GA_3_ treatment developed normally, and corresponding berries grew to reach the size of commercial berries (Figure 1A). Moreover, the vertical and horizontal diameter of ‘Wink’ grape berries treated with GA_3_ increased significantly in comparison to control (Figure 1B).

Accompanied with the formation of seedless berries, the flavor quality of berries affects and leads to important variations. As is well known, total soluble solid is an important indicator of grape berry quality, including sugar, acid, vitamins, and other components [31]. To investigate the effect of GA_3_ on the quality of the grape parthenocarpic seedless berry, we compared total soluble solids, sugar, and titratable acid of berries in GA_3_-treated ‘Wink’ grapes relative to untreated control plants (CK) at the 20, 30, 50, 70, and 95 DAT, respectively (Figure 1C). It was observed that the content of total soluble solids and sugar showed a gradual upward trend accompanied by the ripening of grape berry, and exogenous GA_3_ greatly enhanced their contents at the berries from 50 to 100 DAT. On the contrary, GA_3_ drastically repressed the titratable acid content of grape berry at corresponding stages, and thus the solid acid ratio was significantly increased, leading to a great improvement of ‘Wink’ grape flavor quality.

In general, exogenous GA_3_ treatment efficiently induced grape parthenocarpic seedless berry formation accompanied with the change of berry development and quality, and these variations reflect the potential molecular regulatory roles during the GA-induced parthenocarpy process in grapes.

### 2.2. Identification and Characterization of ARF Family in Grapes

**Figure 2:**
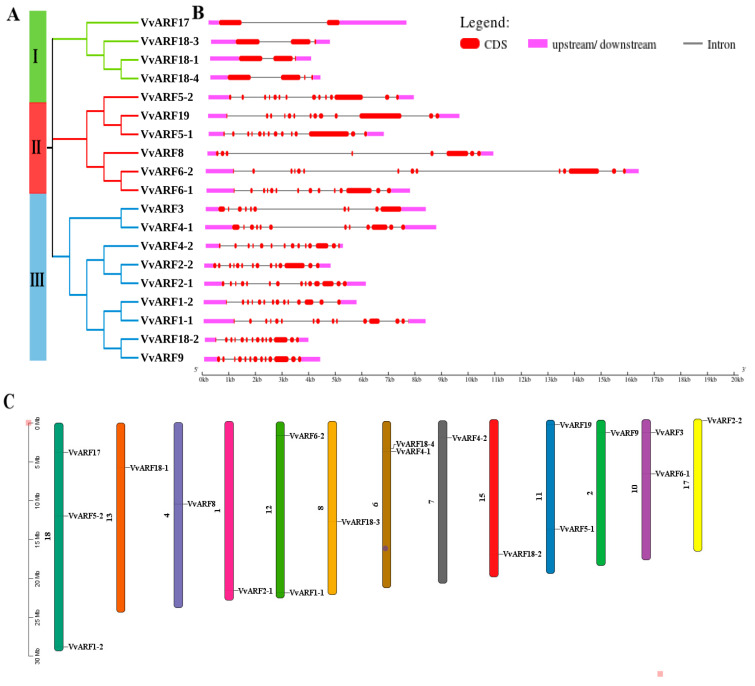
Sequence analysis and chromosomal location of VvARFs. (**A**) The Phylogenetic Treewas generated using MEGA11.0 program by Maximum Likelihood method. (**B**) The exon-intron composition of ARF genes. The coding sequences (CDS) and up- or down-stream regions of ARF genes are represented by red and pink boxes, respectively. Lines represent the introns. (**C**) The chromosome localization of VvARFs.

To recognize the regulatory roles of the VvARF family during GA-induced grape parthenocarpy, we first identified and characterized all members of the ARF family in grape inflorescence tissues during parthenocarpic seedless berry formation. Using RNA-seq data belonging to grape inflorescence during GA-induced parthenocarpy, we identified 19 VvARF members using bioinformatics analysis. Their encoded proteins were further analyzed evolutionarily using MEGA11.0 software. According to the evolutionary tree branch, VvARFs’ member clustering, and gene structure analysis, we classified their results into 3 groups with different members, of which group III had the most members (Figure 2A). Moreover, we analyzed the sequence structure of VvARFs using GSDs online software (Figure 2B) and generally observed the certain similarity in sequence structure belonged to the same group; in group I, the introns of *VvARF* members were short and few, *VvARF17* had the least exons, only 2; while group II showed that the introns of *VvARF* members were long and relatively loose in structure, and *VvARF6-2* had the longest sequence of all genes; Group III showed that the introns of *VvARF* members were short and the structure relatively condensed in structure. Moreover, Map Inspect software was used to locate 19 *VvARF* genes on the chromosomes. The *VvARFs* were distributed in 13 out of 19 grape chromosomes (Figure 2C). The location of *VvARF* members on each chromosome were unequal and there were three *VvARF* members on chr18, two *VvARF* members on chr6, 10, 11, and 12, and only one member on the remaining chromosomes, implying that some members from the ARF family might possess a certain position conservation.

### 2.3. Conservative and Evolutionary Analysis of VvARF Family across Diverse Plant Species

**Figure 3:**
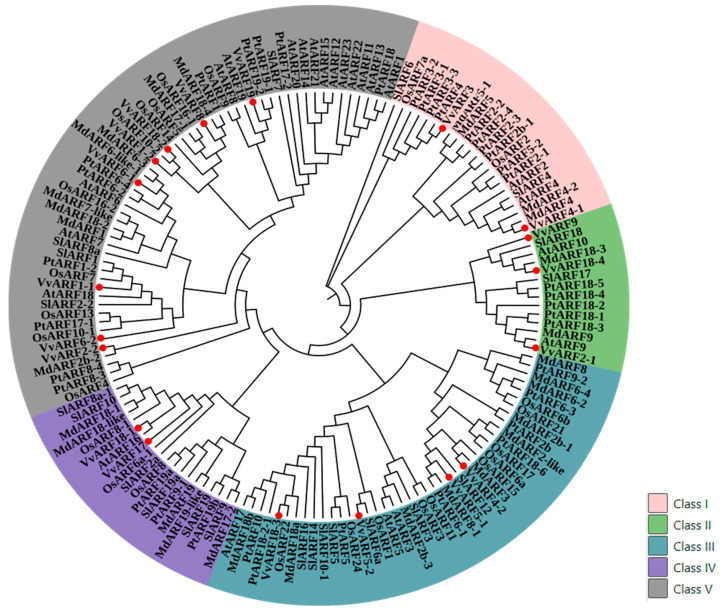
Phylogenetic analysis of ARF family amongsix species. The phylogenetic tree generated after an alignment of deduced *Vitis vinifera*, *Oryza sativa*, *Arabidopsis thaliana*, *Populus trichocarpa*, *Malus domestic* and *Solanum lycopersicum* ARF domains at N-terminus. We constructed the phylogenetic tree using the Maximum Likelihood method against the target species genome and the reference genome. The genes in gene families that were single copies in the whole genome of sequencing species and reference species were used, and the evolutionary tree was constructed using MEGA11.0 to study the evolutionary relationship between species. Based on the results of clustering analysis of homologous gene family, a single copy of homologous gene was selected for multi-sequence alignment (using MUSCLE software for sequence alignment) and then a phylogenetic tree was constructed based on the single copy gene method.

To obtain more intuitive and comprehensive understanding of the genetic relationship of the grape ARF family, MEGA11.0 software was used to perform evolutionary analysis on amino acid sequences from grapes and other five species (*Oryza sativa*, *Arabidopsis thaliana*, *Populus trichocarpa*, *Malus domestic, and Solanum lycopersicum*). The types of *Arabidopsis thaliana* ARF proteins were used to determine the grouping of VvARF proteins; results (Figure 3) show that 154 ARF members were divided into five subgroups, Class I (AtARF2/4-like), Class II (AtARF9/10-like), Class III (AtARF3/5/17-like), Class IV (AtARF16-like) and Class V (AtARF6/7/8/18/19-like). The members of the grape VvARF family are mainly categorized in Class V, with eight members respectively. There are four members in Class III, respectively, while the Class I and IV branch only contains two members. Furthermore, the members of the VvARF family were close to the branches of apple and Arabidopsis, indicated that they have high homology. At the same time, there are five species of ARF genes in these five subgroups, it can be inferred that the ARF protein structure of grapes is relatively conservative.

### 2.4. Screening of Hormone Cis-Elements in the Promoters of VvARFs

**Figure 4:**
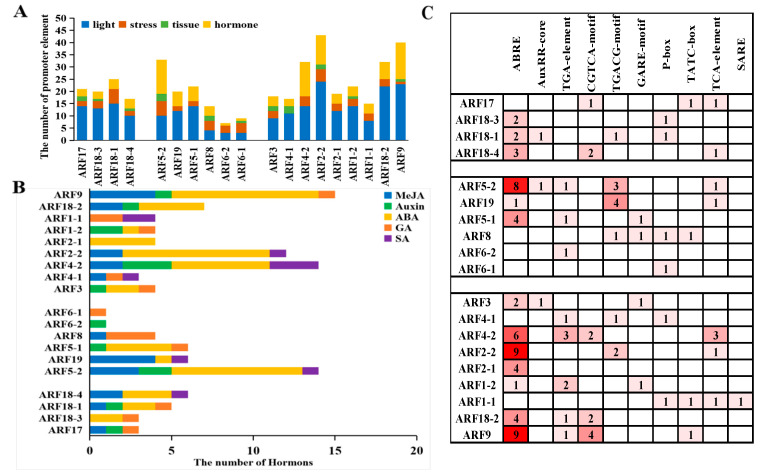
Screening and analysis of cis-elements in the promoters of VvARFs. (**A**): The total number of diverse types of cis-elements derived from VvARFs; (**B**): The number of hormone-responsive cis-elements in promoter regions of VvARFs; (**C**): Types of hormone-responsive cis-elements in promoter regions of VvARFs. ABRE: ABA-responsive cis-elements; AuxRR-core and TGA-element: IAA-responsive cis-elements; CGTCA-motif and TGACG-motif: MeJA-responsive cis-elements; TCA-element and SARE: SA-responsive cis-elements; GARE-motif, P-box and TATC-box: GA-responsive cis-elements.

By analyzing the promoter regions of the VvARF family, we found that their cis-elements can be mainly divided into four categories including: light, tissue-specific, stress-related, and hormone-related responses. Among these four groups, except for light-responsive elements with the highest number, hormone- and stress-responsive elements quantified at the maximum level compared to tissue-specific elements, indicating the VvARF family might have the strongest response to hormones and stresses (Figure 4A). All VvARF family members contained the light- and hormone-responsive elements, which might derive from the fact that these two types of cis-elements are essential for the growth and development of green plants. To further understand the potential roles of VvARF family members in response to hormones, we performed the analysis of the hormone responsive cis-elements in their promoters, and all members of the VvARF family contained hormone response elements, mainly GA-, IAA-, SA-, ABA-, and MeJA (Methyl jasmonate)-responsive cis-elements (Figure 4B). Among them, *VvARF9* had the most hormone-responsive cis-elements, with four, while *VvARF6-1* and *VvARF6-2* had the least, with one hormone-responsive element. The most members of the VvARF family had ABA-responsive cis-elements, and six members of the entire family contained both GA- and IAA-responsive cis-elements (Figure 4C). The analysis of cis-elements implies that the VvARF family might be involved in the crosstalk of multi-hormone signaling to mediate grape growth and development.

### 2.5. Gene Ontology and Pathway Mapping of VvARFs

Based on the analysis of GO and KEGG pathways for the VvARFs family during the GA-induced grape parthenocarpy process, we found that 9 members of the VvARF family were involved in the plant hormone signal transduction (ko04075), participated in tryptophan metabolism pathway (K14486), and affected auxin biosynthesis in plants (Table 1). Among them, there was only 1 member of group I involved in the tryptophan metabolic pathway (K14486), namely VvARF18-1; the members of group II and III participate in the most, respectively, with 4 members. These findings were also supported by GO analysis; it is found that VvARFs have some members involved in the hormone-mediated signaling pathway (GO:0009755), with 6 members, indicating that *VvARFs* might regulate grape parthenocarpy through responding to GA-mediated multi-hormone crosstalk. After GA treatment, out of 19 *VvARFs*, 8 members were up-regulated,10 were down-regulated and only 1 was not expressed in the GA-induced grape parthenocarpy process (Supplementary Table S2), indicating exogenous GA treatment interfered with grape parthenocarpy by inducing the differential expression of *VvARFs*.

**Figure 5:**
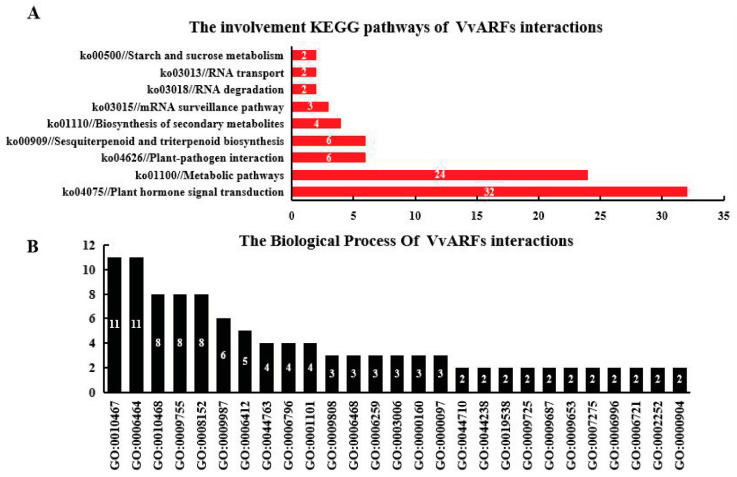
GO and KEEG enrichment analysis of VvARFs family. (**A**): The KEGG pathway annotation of top 9 significant enriched pathways. Numbers on columns: The number of interacted genes involved in KEGG pathway. (**B**): The GO analysis of 27 biological processes. Numbers on columns: The number of interacted genes involved in GO pathway.

To more comprehensively recognize the potential functions of the VvARFs family, we further investigated the molecular interaction, reaction, and relation networks of their interacted proteins. The results showed that a total of 310 interacted proteins were identified from STRING v10.0, of which VvARF4-2 had the most interacted proteins with 179 ones, followed by VvARF18-1 with 122 proteins. Moreover, through KEGG analysis, we revealed that these interacted proteins contributed to 19 KEGG pathways. The pathways with the most interacted proteins were plant hormone signal transduction (ko04075) with 32 members (Figure 5A). Furthermore, these interacting proteins are mainly derived from VvARF4-2, VvARF6-1, and VvARF18-2, indicating that they might be the main regulators of the hormone signaling pathway for grape inflorescence development. Meanwhile, using GO analysis, we found that VvARFs might be involved in 61 biological processes, of which 8 contributed to the hormone-mediated signaling pathway (GO:0009755) (Figure 5B), and these interacted proteins are mainly derived from VvARF6-1 and VvARF9. Interestingly, VvARF6-1 participated in both the hormone-mediated GO and KEGG pathways, indicating it can act as a major factor in the modulation of hormone signaling pathways. After GA treatment, 153 proteins were up-regulated and another 157 were down-regulated, indicating that they might respond to GA-mediated grape parthenocarpy. All genes and their expression levels were listed in Supplementary Table S3.

### 2.6. Differential Expression Profiles of VvARFs and Their Interacted Genes during GA-Induced Grape Parthenocarpy

**Figure 6:**
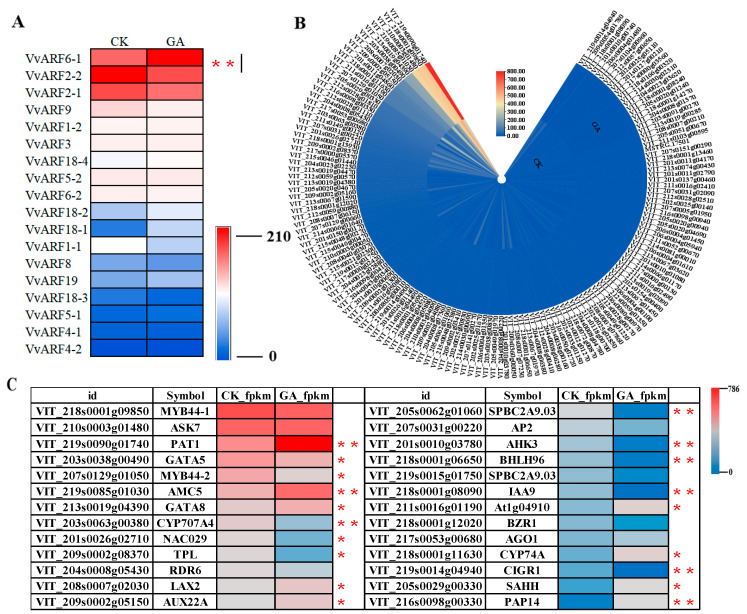
Heat map analysis of VvARFs. (**A**): Heat map analysis of 19 members of grapes VvARF family in response to GA. (**B**): Heat map analysis of 145 genes interacting with the VvARFs family. (**C**): Heat map analysis of genes interacting with the VvARFs family with significantly different expression levels. Asterisks indicate a significant difference between GA and CK by Student’s *t*-test (* *p* < 0.05; ** *p* < 0.01).

Using heat map analysis, we investigated the differential expression profiles of 19 members of the VvARF family during GA-induced grape parthenocarpy. As shown in Figure 6A, in control, *VvARF2-2* had the highest expression level, followed by *VvARF2-1, VvARF6-1,* and *VvARF9*. Meanwhile, *VvARF17* was not expressed, as additionally, *VvARF4-1/4-2/5-1/18-1/18-3* were basically in the low expression levels, while the remaining genes were at a moderate level of expression in the control group. In the treated samples with GA, based on the definition of DEGs [32], out of 19 VvARFs, six members were up-regulated, and another 3 were down-regulated, of which two members (*VvARF18-1* and *VvARF4-2*) were significantly up-regulated, another 4 members (*VvARF6-1, VvARF18-2, VvARF19* and *VvARF5-1*) were slightly up-regulated, conversely, *VvARF18-3*, *VvARF9* and *VvARF1-1* were slightly down-regulated, respectively, while *VvARF17* was not examined in corresponding tissues (Supplementary Table S2; Figure 6A). Notably, of them, the expression level of *VvARF6-1* showed the highest expression with nearly twice that of the control (Supplementary Table S2; Figure 6A). These indicated that VvARF family members responded differently to GA treatment to modulate grape parthenocarpy.

According to the expression profiles of the interacted genes with VvARFs (Figure 6B,C), we revealed that the four genes including *VIT_218s0001g09850* (*VvMYB44-1*), *VIT_219s0090g01740* (*VvPAT1*), *VIT_219s0085g01030* (*VvAMC5*), and *VIT_213s0019g04390*(*VvGATA8*) were highly expressed in the control. During GA-induced parthenocarpy, the expression of *VvPAT1*, *VvAMC5*, and *VvGATA8* increased by twice as much, but that of *VvMYB44-1* was distinctly down-regulated. Therefore, these genes strongly contributed to GA signaling during the modulation of parthenocarpy. As analyzed above, VvPAT1 was predicted to interact with VvARF2-1 and VvARF2-2. Similarly, both VvAMC5 and VvGATA8 were interacted with VvARF4-2 and VvARF18-1, while VvMYB44-1 was associated with VvARF5-1. Therefore, GA also affects the expression of these interacted *VvARF* genes and modulates grape parthenocarpy. On the other hand, we exhibited another 10 interacted genes with *VvARFs* that possess the significant variation at their expression levels during GA-induced grape parthenocarpy, even though they indicated the medium expressions in control. Among them, *VIT_211s0016g01190* (*At1g04910*), *VIT_205s0029g00330* (*VvSAHH*), and *VIT_218s0001g11630 (VvCYP74A)* were up-regulated by GA. On the contrary, *VIT_207s0129g01050 (VvMYB44-2)*, *VIT_203s0063g00380 (VvCYP707A4)*, *VIT_209s0002g08370 (VvTPL)*, *VIT_205s0062g01060 (VvSPBC2A9.03)*, *VIT_201s0010g03780 (VvAHK3)*, *VIT_218s0001g06650 (VvBHLH96),* and *VIT_218s0001g08090(VvIAA9)* were down-regulated in response to GA, while these genes were predicted to mainly interact with VvARF4-2, VvARF6-1, and VvARF18-1, implying that among the VvARF family, they might be the main regulatory factors during GA-induced grape parthenocarpy.

**Figure 7:**
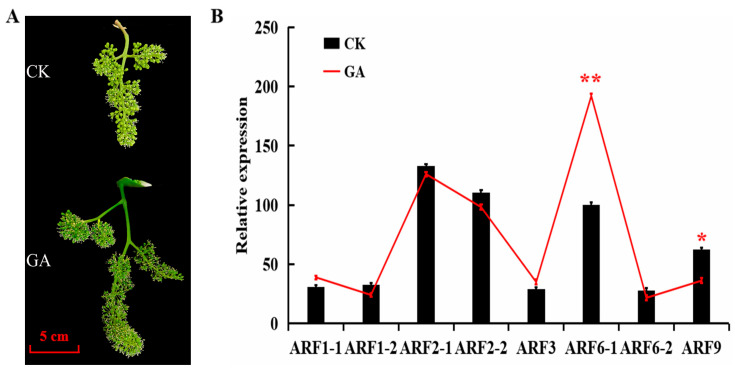
Characterization of VvARFs expression between control and GA treatment at grape flower. (**A**): Inflorescence morphology of grape after GA treatment; (**B**): Expression of VvARF family members in grape flowers. Asterisks indicate a significant difference between GA and CK by Student’s *t*-test (* *p* < 0.05; ** *p* < 0.01).

To validate the result of RNA-seq, 8 *VvARF* genes (*VvARF6-1*, *VvARF1-1*, *VvARF3*, *VvARF6-2*, *VvARF1-2, VvARF9*, *VvARF2-2*, and *VvARF2-1*) were randomly selected to detect their expression levels in corresponding tissues using qRT-PCR. As shown in Figure 7, *VvARF6-1* was up-regulated significantly and *VvARF2-1* was down-regulated by exogenous GA, which were consistent with that in RNA-Seqdata, demonstrating that they might be the important factors of responding to GA-mediated grape parthenocarpy.

### 2.7. Regulatory Network of VvARFs in Response to GA-Mediated Multi-Hormone Signals

**Figure 8:**
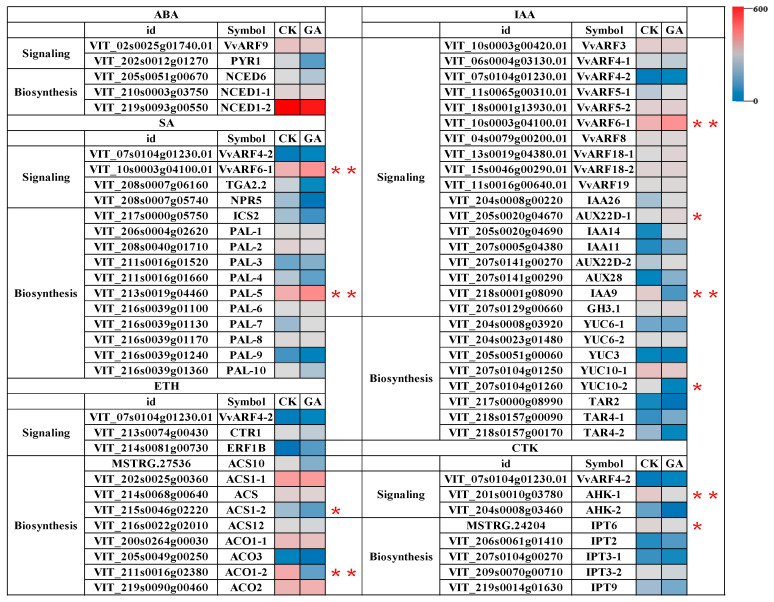
The levels of VvARFs expression and the involvement of hormonal biosynthesis and signaling genes in the plant growth and development. Asterisks indicate a significant difference between GA and CK by Student’s *t*-test (* *p* < 0.05; ** *p* < 0.01).

**Figure 9:**
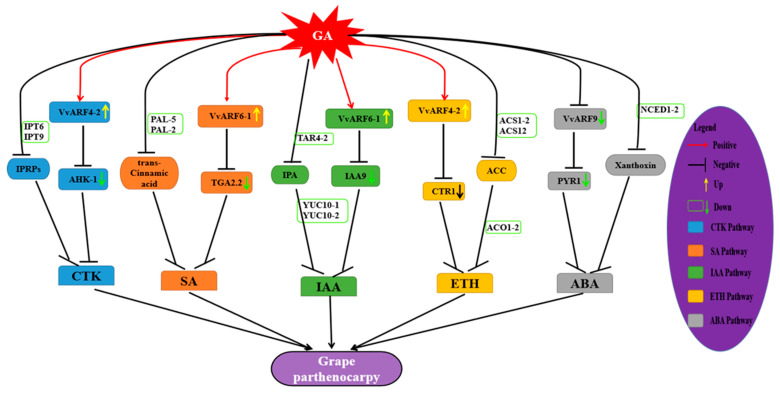
The molecular network of VvARFs mediating plant hormone interaction to regulate plant growth and development. The genes involved in CTK signaling pathway are represented by blue box; The genes involved in SA signaling pathway are represented by orange box; The genes involved in IAA signaling pathway are represented by green box; The genes involved in ETH signaling pathway are represented by yellow box; The genes involved in ABA signaling pathway are represented by grey box.

In this work, we identified 19 VvARFs, and predicted 310 potential interacted genes with VvARFs, of which 32 were involved in the plant hormone signal transduction (ko04075), including IAA, CTK, SA, ABA, and ethylene hormone signaling pathway (Figure 8 and Figure 9). Of them, 10 VvARF members (VvARF3/4-1/4-2/5-1/5-2/6-1/8/18-1/18-2/19) contributed to the auxin signaling pathway through themselves and/or interacting genes (Figure 8). During GA-induced grape parthenocarpy, *VvARF6-1* obviously increased, while the corresponding interacted gene *VvIAA9*, an important repressor in the auxin signaling pathway, reduced. Similarly, *VvYUC10-2*, a key synthesis enzyme in IAA metabolism was down-regulated, and thus we presumed GA promotes the *VvARF6-1* level potentially by inhibiting IAA9, while *VvARF6-1* and *VvYUC10-2* might maintain the dynamic balance of auxin signaling. In the ABA signaling pathway, VvARF9 was predicted to interact with VvPYR1, a receptor protein of the ABA signaling pathway, while *VvPYR1* was down-regulated during GA-induced parthenocarpy, indicating it might possess the negative regulatory effect in ABA signaling; and *VvNCED1-2*, one key ABA synthesis enzyme also decreased. Meanwhile, we revealed that *VvARF4-2* was involved in both the CTK and ETH hormone signaling pathways through interacting genes *VvAHK-1* and *VvCTR1,* respectively, during this process. Exogenous GA repressed the expression of *VvAHK-1* and *VvCTR1* and reduced the levels of *VvIPT* and *VvACS/VvACO1-2* as key enzyme genes in CTK and ETH synthesis. Further analysis demonstrated that both *VvARF4-2* and *VvARF6-1* might participate in the regulation of the SA signaling pathway via interacting with the *VvTGA2.2* gene. Exogenous GA inhibited the expression levels of the *VvTGA2.2* gene and *VvPAL* in the SA synthesis pathway. From these results, we discovered *VvARF4-2* might respond to GA involved in the regulation of 4 hormone signaling pathways including IAA, CTK, SA, and ethylene during parthenocarpy (Figure 8 and Figure 9), followed by *VvARF6-1* that mediates the signaling pathway of GA, auxin, and JA. Therefore, *VvARF4-2* and *VvARF6-1* can be the two key regulatory nodes of the GA-mediated multi-hormone signaling network to modulate grape parthenocarpy.

## 3. Discussion

With the continuous development of biological information technology and the improvement of sequencing technology, it is possible to identify and characterize a certain gene family and its mediating regulatory network at the whole genome level. As a class of ARF genes encoding important transcription factors, they participate in multiple processes of plant growth and development. In this study, based on transcriptome data, 19 ARF genes were identified in the ‘Wink’ grape, which were similar to the ARF family members of *Populus trichocarpa*, *Malus domestic,* and *Solanum lycopersicum*, although the ARF family possessed some evolutionary changes across diverse plant species. ARF family members were divided into 4 till 5 subfamilies, of which the cluster relationship was close to the subfamily members with similar gene functions [19,33,34]. The ARF family gene members of *Vitis vinifera*, *Oryza sativa*, *Arabidopsis thaliana*, *Populus trichocarpa*, *Malus domestic,* and *Solanum lycopersicum* were clustered into 5 branches from Class I to V, of which VvARFs family was distributed in each branch. Both *VvARF6(VvARF6-1/2)* and *AtARF6* were clustered in Class V, implying they might have similar functions. AtARF6 was identified to interact with transcription factors BZR1 and PIF4 in response to GA, BR, IAA, and other hormone in the signaling pathways [35], while BZR1 and PIF4 also specifically interact with AtARF8 to regulate the development of hypocotyls or bud organs. By analyzing the conserved domains of VvARF proteins, it was found that the VvARF gene family has typical ARF conserved domains, B3 domain, Auxin-resp domain, and AUX-IAA domain, which were similar to *Arabidopsis thaliana* [18], rice [19], tobacco [36], tomato [20], longan [37], and sugar beet [38]. Furthermore, the structural distribution and length of exons and introns of *VvARF* family members possess higher conservation with those in *Arabidopsis thaliana* [18], *Oryza sativa* [19], and *Solanum lycopersicum* [20] than the other plant species.

Analysis of VvARF family promoters showed that the cis-acting elements related to hormone responses were identified, of which six VvARF family members contained cis-elements that respond to auxin as well as gibberellin. This implies VvARFs might respond to multi-hormone signal’s crosstalk to be involved in the modulation of plant growth and development, similar to the previous reports on manipulating plant flower development, formation, and bloom [39,40,41], based on the functional conservation of VvARFs and other plant species such as *Arabidopsis thaliana* and *Solanum lycopersicum* described above. In this study, we revealed that *VvARFs* had differential expression profiles in response to GA treatment during grape inflorescence development, of which *VvARF6-1* and *VvARF2-2* showed the higher expression than other members, and up-regulated and down-regulated respectively, might be the important factors of responding to GA-mediated grape parthenocarpy. Together with the analysis of the VvARFs interacted network, we revealed that the *VvARF* family was involved in the regulation of 5 hormone signaling pathways including IAA, CTK, SA, ABA, and ethylene. Of them, *VvARF4-2* might participate with four hormone signaling pathways through interacted genes, and thus might mediate the network of multiple hormone signals to regulate the development of grape flowers. For instance, the arf3 arf4 double mutant *Arabidopsis* plants have narrow petals and deformed stamens, while *Arabidopsis* miR390 positively directs the biosynthesis of trans-acting siRNA3 (tasiRNA3), while tasiRNA3 negatively regulates the expression of *ARF3/ARF4* to affect lateral root development, leaf polarity, and flowering time [42,43,44,45]. In addition, a previous study has shown that silencing *IAA9* gene in tomatoes causes leaf deformation and plant parthenocarpy [46], while *IAA9* could form a complex with ARF8, which prevents *ARF8* from combining with AuxRE region in the promoter of auxin-responsive genes, whereby inhibiting the expression of the auxin response gene and finally preventing fruit setting and development [47], and also induced parthenocarpy in arabidopsis [48]. Similarly, in this study, *VvIAA9* was repressed by exogenous GA, and thus it might participate in the modulation of GA-induced grape parthenocarpy through releasing auxin signaling.

Many research studies confirmed GA was one of the important factors in the efficient induction of plants [6,49,50,51,52], and our previous study also revealed *VvARF10/16/17* were down-regulated during the fruit-set stage of GA-induced grape parthenocarpy; and GA obviously repressed the level of *VvARF18* at the grape seed development period, indicating VvARFs could respond to the GA signal to be involved in manipulation of parthenocarpy. Together with the fact that GA induces plant parthenocarpy through multi-hormones interactions [53,54], we proposed VvARFs might respond to GA-mediated multi-hormones’ interactions to modulate grape parthenocarpy. This view was supported by this work, in which we identified the members responsive to GA of the VvARF family mentioned above and developed their potential regulatory network of responding to GA-mediated multi-hormones’ interactions to regulate grape parthenocarpy (Figure 9). In transgenic tomatoes, the reduced mRNA level of *SlARF7* formed parthenocarpic fruits with the increased auxin and GA responses [55]. Recent research studies suggested *SlARF8a* was up-regulated in parthenocarpy 159-OE tomatoes [11], and the high transcript level of *ARF8* also induced parthenocarpic fruit-set in eggplants independently pollinated/fertilized; while arabidopsis arf6/arf8 flowers arrest as closed buds with short petals, short stamens, and indehiscent anthers, as well as defect in the growth of the pollen tube [28]. Similarly, miR167-OE in tomatoes repressed the expressions of *ARF6/8* to be unable to fruit-set [56]. In this work, we revealed *VvARF6-1* were up-regulated during GA-induced grape parthenocarpy, and it might be involved in modulation of parthenocarpy through responding to both SA and IAA signals. These findings indicate ARFs could participate in modulation of plant parthenocarpic fruit-set.

In addition, we found that many VvARFs might be involved in modulation of hormone synthesis and signals, but most of them have almost no change at their expression levels after GA application, only a few members were distinctly up-/down- regulated by GA, indicating these members might be main factors responsive to GA among the VvARF family. Meanwhile, we could also reveal *VvPAL-5* was up-regulated by GA to promote SA synthesis; conversely, *VvPYR1*, *VvACO1-2*, and *VvYUC10-2* were mainly down-regulated by GA to repress the ABA, ETH, and IAA synthesis, respectively, while genes in CTK synthesis had almost no change after GA application (Figure 8). These results might derive from GA-mediated multi-hormones’ interactions during grape parthenocarpy. As the previous studies [11,57] reported, the auxin signal is at the upstream of the GA signal and it could positively enhance the GA signal; when exogenous GA application was performed, the high GA accumulation could negatively feedback regulate auxin synthesis, similar to the results in this work where exogenous GA application down-regulated the auxin synthesis gene *VvYUC10-2* level, meanwhile high GA content could also boost grape parthenocarpic fruit-set; on the other hand, it was reported that the low ETH content could promote the fruit-set during parthenocarpy [56], which was consistent with the results shown in Figure 8 that exogenous GA repressed the ETH synthesis gene *VvACO1-2* level to boost the fruit-set of parthenocarpic berries. Therefore, GA-mediated multi-hormones’ interactions might be one of the important reasons resulting in grape parthenocarpy.

## 4. Materials and Methods

### 4.1. Plant Materials

Six-year-old Eurasian and diploid grape ‘Wink’ seedlings were selected from the grape experimental base of Jiangsu Academy of Agricultural Sciences. Inflorescences with normal growth and a consistent stage were randomly selected at 5 days before flowering, and dipped in a 50 mg/L GA solution for 30 s. The treated samples with water were used as control. Experiments were performed in three biological replicates. Inflorescence and berry samples were randomly collected from different branches of different treatment groups at different time points [0, 1, 3, 5, 7, 10, 13, 16, 20, 30, 50, 70, and 100 days after treatment (0DAT, 1DAT, 3DAT, 5DAT, 7DAT, 10DAT, 13DAT, 16DAT, 20DAT, 30DAT, 50DAT, 70DAT, and 100DAT respectively). All collected samples were frozen rapidly in liquid nitrogen and stored at −80 °C for further analysis.

### 4.2. Determination of Total Soluble Solids, Sugar and Acidity Content

The horizontal and vertical lengths of the berries were measured using an automatic vernier caliper. Total soluble solids were determined using a portable hand-held dialyzer (PAL-1, ATAGO, Tokyo, Japan), while the content of soluble sugar was assayed according to the anthrone reagent method described by Cao et al. [57]. The titratable acid content was determined by the sodium hydroxide method described by Cao et al. [58]. All experiments were performed with 3 biological replicates. The experimental data were analyzed by Excel, PowerPoint 2019, and GraphPad Prism 8.0.

### 4.3. Phylogenetic Analysis

The software MEGA version 11.0 and ClustalX2 were employed to conduct the phylogenetic analysis. The theoretical gene and protein sequences of grape VvARFs family were obtained from CRIBI database (http://genomes.cribi.unipd.it/grape/index.php, accessed on 1 August 2021). We constructed the phylogenetic tree using the Maximum Likelihood method against the target species genome and the reference genome. The genes in gene families that were single copies in the whole genome of sequencing species and reference species were used, and the evolutionary tree was constructed using MEGA11.0 to study the evolutionary relationship between species. Based on the results of clustering analysis of a homologous gene family, a single copy of homologous gene was selected for multi-sequence alignment (using MUSCLE software for sequence alignment) and then a phylogenetic tree was constructed based on the single copy gene method. Similarly, one more phylogenetic tree was prepared using all the available ARF protein sequences from grapes and the other five species (arabidopsis, rice, apple, poplar, and tomato).

### 4.4. Determination of Exon/Intron Structures of VvARFs

The exon/intron organization of VvARF genes was determined by comparing predicted coding sequences with their corresponding genomic sequences using the GSDS software (http://gsds.cbi.pku.edu.cn, accessed on 8 August 2021).

### 4.5. Analysis of Cis-Acting Elements in Promoter Regions of VvARFs

The sequence of 1500 bp upstream of the transcription initiation site (ATG) of VvARFs was obtained from the grape genome database CRIBI (http://genomes.cribi.unipd.it/grape/index.php, accessed on 16 August 2021) and Grape Genome Browser (http://www.genoscope.cns.fr/externe/GenomeBrowser/Vitis/, accessed on 16 August 2021). For cis-acting element finding, we used PLANTCARE online database (http://bioinformatics.psb.ugent.be/webtools/plantcare/html/, accessed on 16 August 2021).

### 4.6. RNA Extraction, Library Construction and Sequencing

Total RNAs from the diverse samples of GA-treated and control grape ovaries at 5 days post-treatment (anthesis) were extracted using our modified CTAB method [59]. RNA quality was assessed on an Agilent 2100 Bioanalyzer (Agilent Technologies, Palo Alto, CA, USA) and checked using RNase free agarose gel electrophoresis. After total RNA was extracted, eukaryotic mRNA was enriched by Oligo(dT) beads. Then the enriched mRNA was fragmented into short fragments using a fragmentation buffer and conversely transcribed into cDNA by using NEBNext Ultra RNA Library Prep Kit for Illumina (NEB #7530, New England Biolabs, Ipswich, MA, USA). The purified double-stranded cDNA fragments were end repaired, ‘A’ base added, and ligated to Illumina sequencing adapters. The ligation reaction was purified with the AMPure XP Beads (1.0X). Ligated fragments were subjected to size selection by agarose gel electrophoresis and polymerase chain reaction (PCR) amplified. The resulting cDNA library was sequenced using Illumina Novaseq6000 by Gene Denovo Biotechnology Co. (Guangzhou, China).

### 4.7. Analysis of Differentially Expressed Genes

Reads count data was used as input data for the analysis of differentially expressed genes in the software package DESeq2. The analysis was divided into three parts: (1) standardize on read Count; (2) calculate the hypothesis-testing probability (*p*-value) according to the model; and (3) perform multiple hypothesis testing and correction to obtain the false discovery rate (FDR). Based on this analysis, transcripts with FDR < 0.01 and |log2FC| = >1 (where FC is fold-change) were screened as significantly different and classed as differentially expressed genes (DEG). Transcripts with |log2FC| < 0.25 were assumed to no change in expression levels. Other transcripts (0.25 < |log2FC| < 1) were considered as “up-regulated slightly” or “down-regulated slightly”.

### 4.8. Functional Annotation and Enrichment Analysis of Differentially Expressed Genes in VvARFs

Using Kyoto Encyclopedia of Genes and Genomes database (KEGG) (https://www.kegg.jp/kegg/pathway.html, accessed on 7 December 2021) and Gene Ontology (GO) (http://geneontology.org/, accessed on h December 2021), we performed enrichment analysis and unscrambled the interaction network of VvARFs. TBtools was used for heat map analysis.

### 4.9. Predication of Protein-Protein Interaction

The online software STRING (http://string-db.org, accessed on 7 December 2021) was used to predict the interaction proteins of the VvARF family based on our RNA-seq data. For this analysis, we performed as follows: Select “multiple proteins “, input VvARF family member gene number at “the protein name”; enter Vitaceae at “Organism”; then, the results of the interacted proteins for VvARFs were predicted in this work.

### 4.10. Quantitative Real-Time Polymerase Chain Reaction (qRT-PCR) Analysis

To evaluate the reliability of the sequencing results, 8 VvARFs were selected randomly for qRT-PCR verification. The *Actin* gene was used as a reference gene in the qRT-PCR detection of VvARFs. The primers were presented in Supplementary Table S1. The RNA of grape flowers was extracted by CTAB method [59]. For the amplification system and procedure, we used the SYBR^®^ Premix Ex TaqTM II kit from TaKaRa Company according to the instructions. All cDNAs were stored at −80 °C for the next analysis. All experiments were performed with 3 biological replicates and relative expression was calculated according to the 2^−ΔΔCt^ method.

## 5. Conclusions

In summary, a total of 19 VvARF members were identified in the ovaries during GA-induced grapes’ parthenocarpy, and almost all VvARF members were GA-responsive genes, of which 8 members were up-regulated, and another 10 were down-regulated by exogenous GA. Furthermore, VvARFs were predicted to have 310 interacted proteins involved in the 19 KEGG pathways and participated in IAA, ABA, CTK, SA, and ethylene signaling pathways by responding to GA-mediated multi-hormone crosstalk. *VvARF4-2* might be the major factor in the modulation of GA-induced parthenocarpy via the crosstalk of IAA, CTK, SA, and ethylene signaling, followed by *VvAFR6-1* and *VvARF9* involved in SA and ABA signaling pathways, respectively. These findings provide novel insight into the GA-mediated multi-hormone signaling networks to regulate the parthenocarpy process via guiding the VvARF family, which has implications for the molecular breeding of high-quality seedless grape berries.

## Figures and Tables

**Table 1:** GO and KEGG pathway of VvARFs.

	Gene ID	Symbol	Pathway	K_ID	GO Process
I	VIT_18s0001g04180	VvARF17			GO:0009987//cellular process
	VIT_208s0040g01810	VvARF18-3			GO:0009755//hormone-mediated signaling pathway
	VIT_213s0019g04380	VvARF18-1	ko04075//Plant hormone signal transduction	K14486	GO:0007275//multicellular organism development
	VIT_206s0004g02750	VvARF18-4			GO:0009718//anthocyanin-containing compound biosynthetic process
II	VIT_218s0001g13930	VvARF5-2	ko04075//Plant hormone signal transduction	K14486	GO:0000578//embryonic axis specification
	VIT_211s0016g00640	VvARF19	ko04075//Plant hormone signal transduction	K14486	GO:0009755//hormone-mediated signaling pathway
	VIT_211s0065g00310	VvARF5-1	ko04075//Plant hormone signal transduction	K14486	GO:0009755//hormone-mediated signaling pathway
	VIT_204s0079g00200	VvARF8			-
	VIT_212s0028g01170	VvARF6-2	ko04075//Plant hormone signal transduction	K14486	GO:0001101//response to acid chemical
	VIT_210s0003g04100	VvARF6-1			GO:0003002//regionalization
III	VIT_210s0003g00420	VvARF3	ko04075//Plant hormone signal transduction	K14486	GO:0001708//cell fate specification
	VIT_206s0004g03130	VvARF4-1			GO:0001708//cell fate specification
	VIT_207s0104g01230	VvARF4-2			GO:0009987//cellular process
	VIT_217s0000g00320	VvARF2-2			GO:0001101//response to acid chemical
	VIT_201s0244g00150	VvARF2-1			GO:0006355//regulation of transcription, DNA-templated
	VIT_218s0089g00910	VvARF1-2	ko04075//Plant hormone signal transduction	K14486	GO:0009755//hormone-mediated signaling pathway
	VIT_212s0035g01800	VvARF1-1	ko04075//Plant hormone signal transduction	K14486	GO:0009755//hormone-mediated signaling pathway
	VIT_215s0046g00290	VvARF18-2			GO:0009725//response to hormone
	VIT_202s0025g01740	VvARF9	ko04075//Plant hormone signal transduction	K14486	GO:0009755//hormone-mediated signaling pathway

## Data Availability

Not applicable.

## Supplementary Materials

The following supporting information can be downloaded at: https://www.mdpi.com/article/10.3390/ijms231911108/s1.
