# Supplementary material for: Identification and Characterization of AUXIN Response Factor Gene Family Reveals Their Regulatory Network to Respond the Multi-Hormones Crosstalk during GA-Induced Grape Parthenocarpic Berry
Source: Int J Mol Sci. 2022 Sep 21;23(19):11108. doi: 10.3390/ijms231911108 (PMC9569648; doi:10.3390/ijms231911108)
Supplement: Supplementary file 1 [file ijms-23-11108-s001.zip › Supplementary Table S1.pdf]

Table S1 Sequences and use of primers

Name	Forward primer sequences (5’-3’)	Reverse primer sequences (5’-3’)	Use
**VvActin**	**GCTCGCTGTTTTGCAGTTCTAC**	**AACATAGGTGAGGCCGCACTT**	**qRT-PCR**
**VvARF1-1**	**ACCGGAACCCTCTTTTCTGT**	**ACCAACAATTGTGCCACTGA**	**qRT-PCR**
**VvARF1-2**	**AAGGGCTGTGGATTTGACAC**	**AACTCATGCCAAGGGTCATC**	**qRT-PCR**
**VvARF2-1**	**CGACTCCTGCAAATGTCAGA**	**GTGGCTTTAACCCCATGAGA**	**qRT-PCR**
**VvARF2-2**	**CCGGGGATGCCTTTATATTT**	**ACTCTGCAGGGCTTGTCCTA**	**qRT-PCR**
**VvARF3**	**GCTGCAGAGGACTGTTTTCC**	**TTTGAGCTGCTCTTCGGATT**	**qRT-PCR**
**VvARF6-1**	**AGCAACATTTGCAGCATCAG**	**TCCCTGGGTTACCAGTTGAG**	**qRT-PCR**
**VvARF6-2**	**CTCACATTGGCGCTCTGTAA**	**CTGAATCCCCTGATCTCCAA**	**qRT-PCR**
**VvARF9**	**AGGCTATTCAACGCTGCACT**	**CGCTCGAAACACTGATTGAA**	**qRT-PCR**
